# Assessing global pandemic risks from emerging infectious diseases and high containment laboratory leaks: A country‐level spatial network SIR model analysis

**DOI:** 10.1111/risa.70070

**Published:** 2025-10-30

**Authors:** Ross J. Tieman, Pedro Adami Oliboni, Simeon Campos

**Affiliations:** ^1^ Alliance to Feed the Earth in Disasters (ALLFED) Lafayette Colorado USA; ^2^ Fenner School of Environment & Society Australian National University Canberra Australian Capital Territory Australia; ^3^ Department of Economics University of Chicago Chicago Illinois USA

**Keywords:** global catastrophic risk, pandemics, SIR model

## Abstract

Future pandemics could arise from several sources, notably, emerging infectious diseases (EID); and lab leaks from high containment biological laboratories (HCBL). Recent advances in infectious disease, information technology, and biotechnology provide building blocks to reduce pandemic risk if deployed intelligently. However, the global nature of infectious diseases, distribution of HCBLs, and increasing complexity of transmission dynamics due to travel networks make it difficult to determine how to best deploy mitigation efforts. Increasing understanding of the risk landscape posed by EID and HCBL lab leaks could improve risk reduction efforts. The presented paper develops a country‐level spatial network susceptible‐infected‐removed model based on global travel network data and relative risk measures of potential origin sources, EID, and lab leaks from biological safety level 3+ and 4 labs, to explore expected infections over the first 30 days of a pandemic. Model outputs indicate that EID and lab leaks in India, the USA, and China are most impacted at day 30. For EID, expected infections shift from high EID origin potential countries at day 10 to the USA, India, and China, while for lab leaks, the USA and India start with high lab leak potential. With respect to model uncertainties and limitations, results indicate several large, wealthy countries are influential to pandemic risk from both EID and lab leaks, indicating high leverage points for mitigation efforts.

## 1 INTRODUCTION

This paper assesses the role of global travel networks in propagating risk from two origin sources for pandemic potential pathogens (PPP): emerging infectious disease (EID), and lab leaks, through the development of a spatial network susceptible infectious removed (SIR) model. Combining network analysis with origin risk assessment reveals dynamics that neither approach captures alone.

Infectious diseases have historically caused enormous human suffering, from the Black Death (200M deaths) (Perry & Fetherston, 1997) to the Spanish flu (17.4–50M deaths, 0.95–2.7% of global population) (Spreeuwenberg et al., 2018) (Johnson & Mueller, 2002). Even with modern medicine, COVID‐19 demonstrated our continued vulnerability to pandemics, which are considered potential existential risks. Estimates of natural pandemic existential risk range from 1/2000 to 1/20,000 per century, though these figures warrant skepticism due to methodological limitations (Millett & Snyder‐Beattie, 2017; Sandberg & Bostrom, 2008). Moreover, infectious disease risk increases significantly with engineered pathogens, estimated at approximately 1/30 per century (Ord, 2020).

Historically, transmission of novel pathogens from animals to humans has been the major source of EID and, consequently, pandemic risk. More recently, human interaction with PPP in laboratory settings for purposes such as diagnostic testing, vaccine development, and the development of biological weapons has yielded a new reservoir of PPP. Though most work with PPP is undertaken in designated Biological Safety Level (BSL) 3 and 4 laboratories, highly secure laboratory facilities specially developed to prevent high‐risk pathogens from being released into the environment, many records of pathogen escape, henceforth referred to as “lab leaks”, from BSL 3 and 4 facilities exist. Notable examples of lab leaks include: The 2007 UK outbreak of Foot and Mouth Disease from the Pirbright facility, a BSL 4 lab (DEFRA, 2008), and Venezuelan equine encephalitis virus in 1995, which was identical to a lab strain from 1963 (Brault et al., 2001). Consequently, BSL 3 & 4 labs are a significant source of pathogen risk, arguably of higher concern than EID due to by‐design selecting for high‐risk pathogens and being located in urban areas (Merler et al., 2013).

Global air travel networks facilitate rapid dispersion of pathogens (Holmes et al., 2018), further increasing the risk posed by the emergence of new diseases or lab‐leaked pathogens. Even if a particular country is not likely to give rise to a new emerging infectious disease or be the source of a lab leak, it might be strongly connected to other countries that do face such a risk.

Reducing the risk of harm from pandemics should be considered a high priority (Millett & Snyder‐Beattie, 2017). Therefore, it is important to understand which countries most influence global pandemic risk and how to use this information to prioritize risk mitigation efforts. By integrating global travel networks into our risk assessment, we reveal a pandemic risk landscape that is different from what is observed when looking solely at the risk of origin. This network perspective exposes how countries with minimal origin risk may still have significant influence on pandemic risk through their connections to high‐risk regions. This can help identify high‐leverage locations for interventions, such as screening of people who present symptoms that cannot be diagnosed easily (Holmes et al., 2018).

This paper contributes to the understanding of pandemic risk by investigating the interaction of travel networks with two sources of PPP, EID and lab leaks from BSL 3+ and 4 labs. We use a spatial network SIR model informed by the relative distribution of risk of outbreak from two potential sources of PPP (EID and lab leaks) to study how risk propagates through travel networks. To our knowledge, there has been no attempt to aggregate origin risk from EID and lab leaks with travel networks in assessing pandemic risk. Overlaying the risk distribution of EID and lab leak sources can shed light on commonalities in risk propagation between different origin sources to better target intervention deployment.

The presented model provides a coarse‐grained picture of pandemic risk that captures travel network influence on origin risk. We hope that our model and associated findings motivate future work to reduce the uncertainties faced in understanding the highly complex phenomena involved in pandemic risk.

## 2 METHODS

To investigate the role of travel networks, a SIR spatial network model using countries as nodes was developed, where the network is derived from travel data. The model is run with each country (*n* = 195) as a pandemic origin source for a set of disease parameters. Outputs of the model are then scaled according to the relative risk of origin in a given country. The origin sources are EID or lab leak. The SIR model structure, method of aggregation, and EID and lab leak origin potential measures are described in more detail below.

### 2.1 SIR model overview

The model is an adaptation of the SIR network model over cities described by Muroya et al. (2013), chosen as inspiration due to its simplicity. The model considers the world population 𝑁, partitioned into 𝑛 countries indexed by *i *= 1,2,…,𝑛. The population within the *i*th country (*Ni*) is partitioned into susceptibles (𝑆*i*), infectives (𝐼*i*), and removed (recovered and dead) (𝑅*i*).

Equation (1) that describes the system is as follows:

(1) $$\frac{dS_{i}}{dt}=-S_{i}\sum_{j\neq i}m_{ij}-S_{i}\beta_{i}\frac{I_{i}}{N_{i}}+\sum_{j\neq i}m_{ji}S_{j}.$$

In words, the change in 𝑆𝑖 depends on how many susceptibles migrate out, how many susceptibles get infected, and how many susceptibles from other countries migrate in (Equation 2).

(2) $$\frac{dI_{i}}{dt}=S_{i}\beta_{i}\frac{I_{i}}{N_{i}}-I_{i}\gamma_{i}-I_{i}\sum_{j\neq i}m_{ij}+\sum_{j\neq i}m_{ji}I_{j}.$$

In words, the change in 𝐼𝑖 depends on how many susceptibles get infected, how many infectives are removed^1^, how many infectives migrate out, and how many infectives from other countries migrate in (Equation 3).

(3) $$\frac{dR_{i}}{dt}=\gamma_{i}I_{i}-R_{i}\sum_{j\neq i}m_{ij}+\sum_{j\neq i}m_{ji}R_{j}.$$

In words, the change in 𝑅𝑖 depends on how many infectives are removed, how many are removed and migrate out, and how many are removed from other countries and migrate in.

Notation: $m_{ij}$ is the rate of travel from 𝑖 to 𝑗 at each time step; $\gamma_{i}$ is the rate at which infected people in country 𝑖 are removed from the disease pool; $\beta_{i}$ is the transmission rate, which indicates the rate at which $S_{i}$ individuals are infected by each individual in $I_{i}$.

This is a minimal model that allows for the study of indirect connections (travel from country A to B to C) and the variation of disease characteristics.

The global spatial network SIR structure models infectious disease dynamics as a phenomenon that happens in a geopolitical network with countries as nodes and travel flows as directed links. The crucial simplification this model makes is of abstracting away from the network of people interactions to a network of country interactions that are mediated by flows of people. This abstraction merits careful consideration regarding lab leaks, as it averages out the influence of population density and co‐location of BSL labs in urban centers (see discussion).

Network SIR models with cities as nodes are common in the literature (Muroya et al., 2013; Pujari & Shekatkar, 2020). But to our knowledge, there are no other SIR‐like network models for global disease spread that use EID and lab leaks as a source of pathogen origin. The most similar models in terms of level of abstraction use networks of states inside a country, such as Sharma et al. (2021), who develop a SEIRD model (an elaboration of the SIR model) over a network of states in India (which are larger in terms of population than many countries).

#### 2.1.1 Global travel data

The Global Transnational Mobility dataset is used to develop the network characteristics (Recchi et al., 2020). This dataset describes how many “trips” there have been between any two countries in the world from the years 2011 to 2016 (1 trip = 1 person traveling)^2^. These estimates of trips account for multiple modes of transport and have been put together by aggregating global statistics on tourism and air passenger traffic (Recchi et al., 2019). The dataset reports travel as trips per year, which we converted to average daily trips in order to obtain a finer‐grained time scale.

#### 2.1.2 SIR disease parameters

The model uses transmission and removal rate parameters ($\beta_{i}$ and $\gamma_{i}$). Transmission and removal rates are also assumed to be the same for all countries. For simplicity, a single set of disease parameters equivalent to SARS‐CoV‐2 is used for all countries for both EID and lab leaks (see Section 4). This maintains focus on the geographic, regulatory, and socioeconomic factors captured in origin potential measures and the role of travel networks, and avoids determining plausible distributions of disease parameters likely to arise from different origin sources, which would be an extensive piece of work. Future iterations of the model could explore distributions of disease parameters equivalent to anticipated PPP informed by historical data^3^.

SARS‐CoV‐2 parameter values used are from Amiri Mehra et al. (2020) (Table 1). It should be noted that parameter values for a given disease are influenced by many factors not captured within the model, so although informed by empirical data, the parameters are ultimately abstractions.

**TABLE 1:** SIR model parameters for SARS‐CoV‐2 (COVID‐19) calculated by Amiri Mehra et al. (2020).

Parameter	β(transmission rate) ${\left(\mathrm{person}.\mathrm{day}\right)}^{-1}$	γ(removal rate) ${\left(\mathrm{day}\right)}^{-1}$
Value	1	0.223

The network SIR model used in this study is deterministic, meaning that identical initial conditions and parameters produce identical outcomes. For each country with pandemic origin potential (EID or lab leak), the model is run once with an initial outbreak of 1000 infected individuals originating in that country. Each model run consists of 30 days. A country has origin potential if it has an EID vulnerability score in Moore et al. (2017) or has at least one lab as reported by the Global Biolabs Project (Koblentz et al., 2021; Moore et al., 2017). Due to the model's deterministic nature, each country‐origin scenario is run once, as multiple runs would produce identical results. To test result robustness, we conducted sensitivity analyses using alternative parameter sets and country inclusion criteria, detailed in the results section.

### 2.2 Relative risk of pandemic origin

Considering all countries *C*. Given a pandemic being initiated, the probability that the pandemic originated in a given country *c* is defined using Equation (4):

(4) $$Pr\left(c\right)=\frac{r_{c}}{\sum_{r\in C}r_{c}^{\prime }},$$

where $r_{c}$ is the origin source potential of country *c*. Origin potential measures the relative strength of contributing factors to a pandemic being initiated in a given country.

Relative risk of pandemic origin is considered separately for EID and lab leaks.

#### 2.2.1 Emerging infectious disease origin potential

The origin potential of EID is based on the infectious disease vulnerability index developed in Moore et al. (2017). The Infectious Disease Vulnerability Index aims to identify countries that are most vulnerable to outbreaks of infectious diseases with potential for transnational spread to inform preemptive actions that mitigate the spread and consequences of such transnational infectious disease outbreaks (Moore et al., 2017). The Vulnerability Index is derived from a variety of factors in the following seven domains: demographic, public health, economic, disease dynamics, health care, political–domestic, political–international. These factors are then weighted according to elicited expert opinions.

Vulnerability scores in Moore et al. (2017) were transformed by subtracting the score from 1. This is because the original scores are out of [0,1], where a lower score means a country is more at risk. But for the purpose of this model, a country that is more at risk should be given a higher rather than a lower weight.

In particular, to Moore et al. (2017), it is important to note that migration (average annual number of migrants per 1000 people) is part of the vulnerability score. So travel has been included in some (albeit relatively minor) way in their assessment.

#### 2.2.2 Lab leak origin potential

A simple measure of lab leak origin potential from BSL 3+ and 4 labs for a given country is given by Equation (5):

(5) $$r_{c}=b_{c}\sum_{i=1\;}^{n_{c}}a_{ic},$$

which can be determined from the following information:

1. The number of BSL‐4 and BSL‐3+ labs in each country, $n_{c}$.
2. Area of BSL‐4 labs and BSL‐3+ in each country, $a_{ic}$.
3. Global biolabs biorisk management scorecard (GBBMS) (Koblentz et al., 2021) score for each country, $b_{c}$.

Data for 1–3 are provided by the Global Biolabs Project (GBP), which represents the most developed dataset on BSL 3+ and 4 locations^4^^,^ 5 (Koblentz et al., 2021, 2023). Missing data required for 1–3 were inferred by the following actions:

- BSL 4 lab entries without lab area data were assigned the geometric mean of available BSL 4 lab area data.^6^ BSL3+ lab entries that do not include the area were assigned this value as well.
- Countries with a lab but no GBBMS score were assigned the arithmetic mean of all GBBMS scores.

BSL3+ numbers provided by GBP roughly match BSL 3 labs reported in *Mapping Biosafety Level‐3 Laboratories by Publications* (Schuerger et al., 2022), which reports 57 BSL 3 locations globally, while GBP reports 55 active BSL 3+ labs. The closeness of the two numbers is somewhat suspicious, given that BSL3+ is a higher classification of 3; intuitively, one would expect a significantly larger number of reported BSL 3 labs. The distribution of BSL 3 labs reported by Schuerger et al. (2022) and BSL 3+ labs by Koblentz et al. (2021) do not match at a continental level. Discrepancies between numbers might be explained by differences in method. Schuerger et al.’s (2022) estimate uses PubMed publications and would miss nonpublishing labs, such as diagnostic labs. The lack of standardization of BSL level characterization may also impact lab numbers and characterization (see Section 4).

The GBBMS score is incorporated into the lab leak potential measure to indicate how comparatively risky labs in a given country are. The GBBMS is out of 48 biosafety management indicators derived from standards and best practices endorsed by WHO, ISO 35001, NTI, and other organizations. Indicators are scored positive if relevant statutory legislation (regulations, standards, and policies) is present in a country. The metric does not capture compliance or enforcement, decreasing its robustness.

The developed lab leak measure assumes that the risk of lab escape is proportional to the lab area. This is based on the intuition that greater lab area implies a greater number of lab experiments and required supporting systems, leading to greater potential for human error (Wurtz et al., 2016), mechanical failure (DEFRA, 2008), or other failures resulting in pathogen escape. For this version of the model, a linear relation between lab area and risk is assumed.^7^ There is uncertainty in this assumption, for instance, an argument based on economies of scale would imply that bigger labs can afford better infrastructure, thus reducing risk contribution per unit area. A more in‐depth investigation of the relationship between lab leaks and lab size is deferred to future work.

## 3 RESULTS

Model results are aggregated across all simulations. That is, we average over all countries as origin sources to obtain an “average” number of cumulative infections^8^, henceforth abbreviated to ACIs for each country. This is performed for the two considered pandemic initiation sources (EID or lab leak). Each model run provides the number of susceptible, infected, and removed in each country in the world for the first 30 days after the initial outbreak. Results of the SIR model are averaged independently for EID and lab leak risk as the relative risk scores (see Section 2.2) use different underlying measures that are not directly comparable. To average model results, the relative risk of pandemic origin for each country is multiplied by the results of the model run that had the country as the origin of the pandemic, and the products are then summed.

This type of averaging approach gives a measure of relative risk to early‐stage pandemics. One interpretation of this measure is that it provides an estimate of the expected number of cumulative infections in each country, conditional on a pandemic happening with a distribution of likelihood of pandemic origin from the considered origin source (EID or lab leaks) described in Section 2.2.

Other methods of aggregating and interpreting the results from this type of model are possible and would be valuable to explore in future work. It is clear that average cumulative infections, as defined in this paper, will not be the appropriate risk measure for many important prioritization problems. Different decision problems may ask for different risk measures. One may be interested, for instance, in preventing the largest number of countries from reaching a certain threshold of cases in an early‐stage pandemic. In this case, it could be that a measure that gave weights to each model run based on this threshold would be more appropriate, for instance. Still, the risk measure of average cumulative infections does provide a general picture of risk from early pandemics that already begins to capture how travel networks affect the pandemic risk distribution.

Before presenting the specific findings for EID and lab leak scenarios, we note two sensitivity analyses that inform the interpretation of all results. First, we tested an alternative parameter set (β = 3, γ = 0.1, with 500 initial infections) to examine the model's sensitivity to disease characteristics. The rank‐biased overlap^9^ (RBO) between the two parameter sets for country rankings at 30 days was 0.54, with 24 countries appearing in both top‐30 lists (see Appendix 2, Table A2‐5). Second, we analyzed sensitivity to country size by excluding countries with populations below 5 million from consideration as origin points. This yielded a rank‐biased overlap of 0.78 between the full and restricted country sets (see Appendix 1, Table A1‐1). These analyses suggest that while specific country rankings are somewhat sensitive to parameter choices, the broad patterns of risk distribution remain consistent across different scenarios. Detailed results from both sensitivity analyses are provided in Appendices 1 and 2.

Results from the aggregation described above can be illustrated with heatmaps of cumulative infections at 10, 20, and 30 days after the initial outbreak. In these heatmaps, color intensity represents each country's proportion of global aggregate cumulative infections (ACI); for example, a country with a proportion of ACI of 0.05 would have 5% of the global total aggregate cumulative infections at the considered timestep; EID and lab leak results are explored separately in the following sections.

### 3.1 EID model results

**FIGURE 1:**
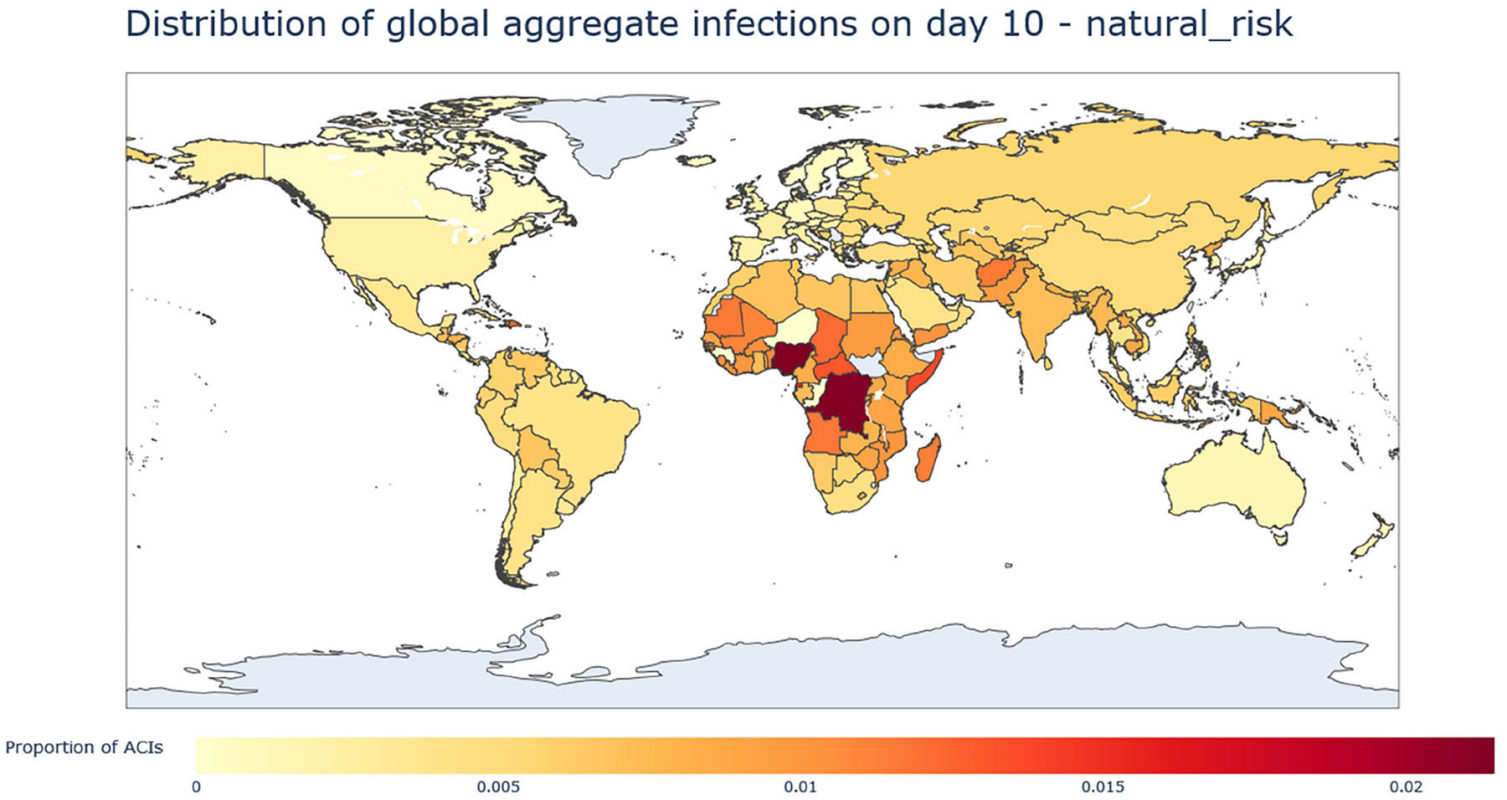
Heatmap of the distribution of ACIs from EID at day 10, measured as proportion of ACIs. The map shows the concentration of ACIs in Central African Countries, notably Nigeria, the Democratic Republic of Congo, Equatorial Guinea, and the Central African Republic.

**FIGURE 2:**
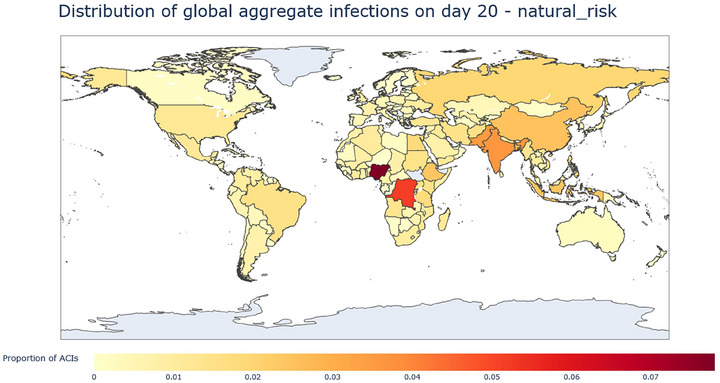
Heatmap of the distribution of ACIs from EID at day 20, measured as proportion of ACIs. The map shows a shift in the concentration of ACIs from Central Africa toward Asia.

**FIGURE 3:**
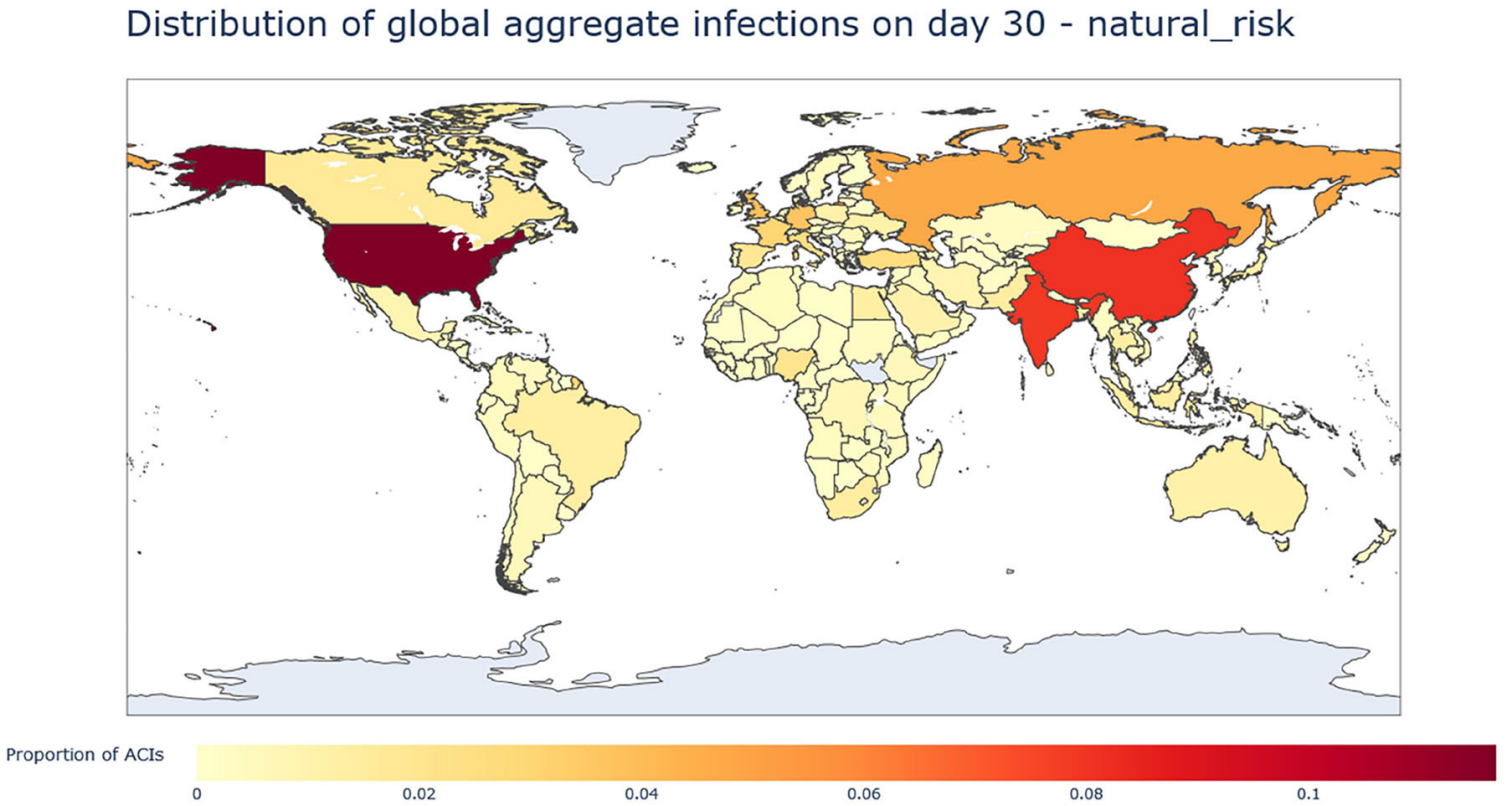
Heatmap of the distribution of ACIs from EID at day 30, measured as proportion of ACIs. The map shows a major jump in the proportion of ACIs located in the USA and continued growth in India and China.

Figures 1, 2, 3 demonstrate a shift in the concentration of ACIs from high EID origin potential countries (Nigeria, Democratic Republic of Congo, Equatorial Guinea, Central African Republic) to important travel nodes. At 10 days (Figure 1), the distribution of ACIs is largely concentrated in Africa, which reflects EID origin potential scores. After 20 days, we see that in Africa, ACI concentrates in Nigeria and the DRC, which already stood out after 10 days, and ACIs also start to spread more intensely in Asia. Interestingly, at 30 days, the picture changes significantly. The USA rises as the country with the largest number of average cumulative infections in the world, Africa bears much less of the world's share of cumulative infections (although Nigeria is itself still prominent), and India, China, Russia, and Europe concentrate much of the rest of the ACIs.

We would not expect that the average numbers would closely match the spread of a specific disease originating in a particular country, but at the run‐level, the model shows notable alignment with SARS‐CoV‐2 when accounting for unreported infections. At 30 days, the model's average cumulative infections (ACI) in the United States are about 30 million. However, when examining the model run with China as the country of origin, after 30 days, there are approximately 5 million cumulative cases in the US. This initially appears to diverge from real‐world observations, where it took about 150 days for confirmed cases to reach this number (Johns Hopkins Coronavirus Resource Center, n.d.). However, when estimated unreported instances are considered, the model results more closely approximate the actual spread of SARS‐CoV‐2. Indeed, studies estimated approximately 5 million cumulative cases in the USA by April 4, 2020, which is about one month after the 50th case was reported in the USA (Lu et al., 2021).

**FIGURE 4:**
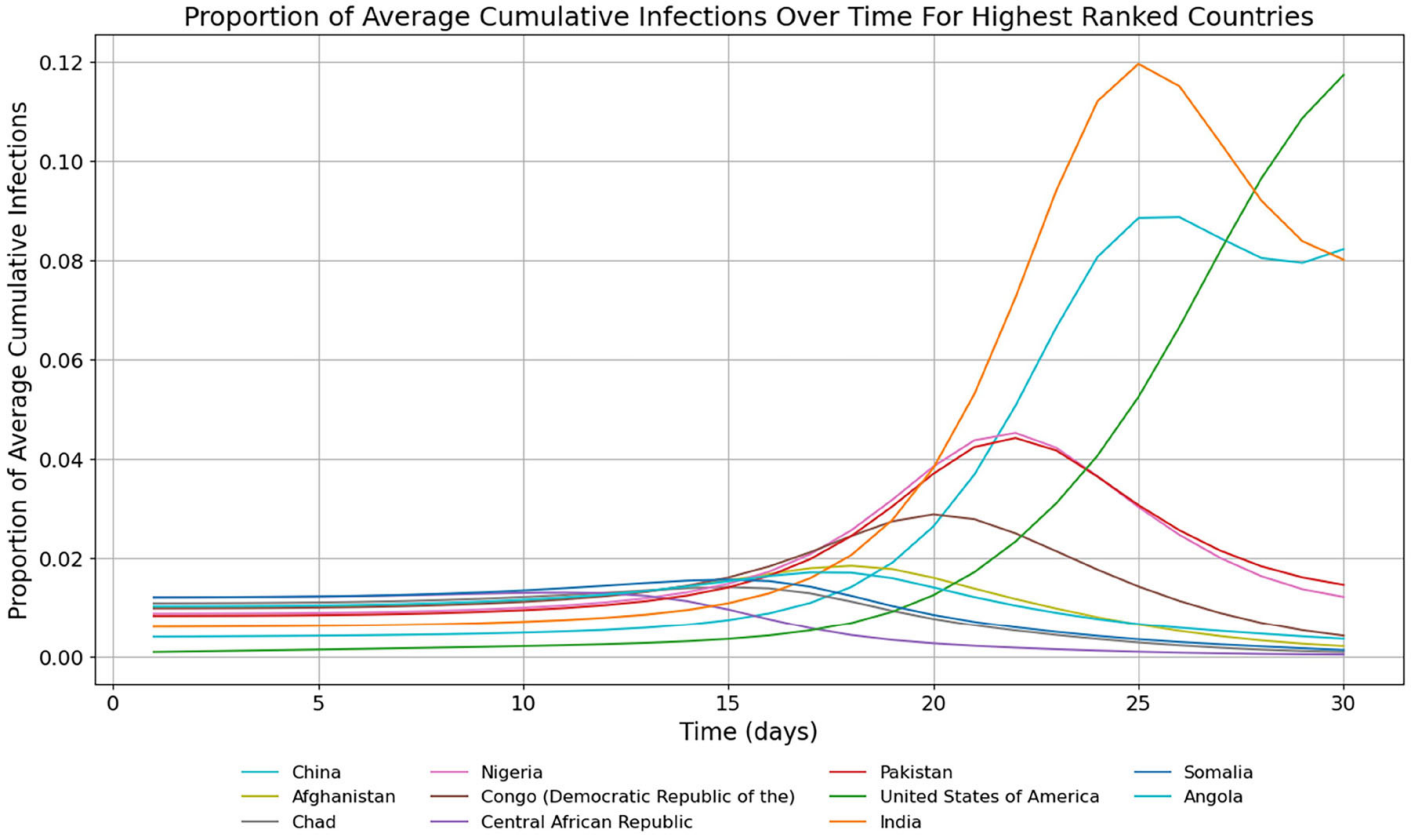
Proportion of ACIs by top‐ranking countries from days 1–30. Only countries in the top 3 of ACIs at any time between day 1 and 30 are shown. The USA, China, and India have the highest proportion of ACIs at day 30.

**FIGURE 5:**
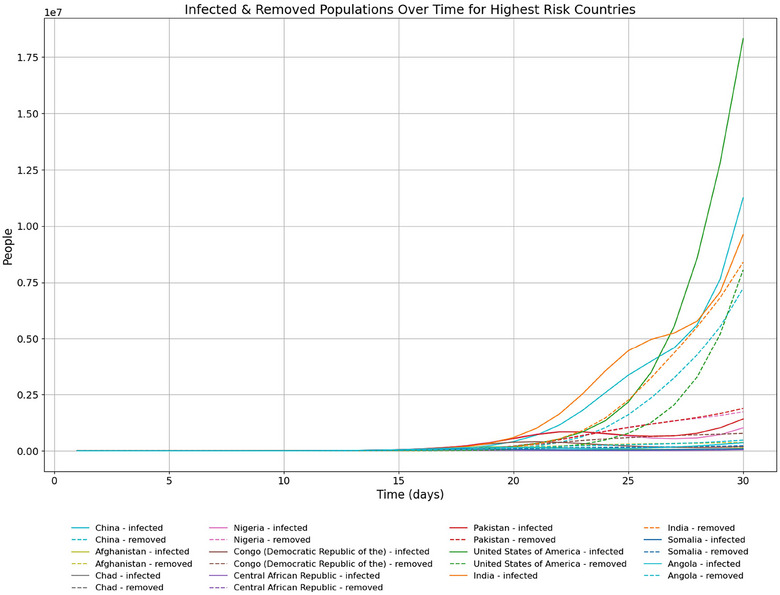
Time series of infected and removed population for the highest risk countries by proportion of ACIs. Only countries in the top 3 of ACIs at any time between days 1 and 30 are shown.

In terms of proportion of ACIs, time series of model results show the fast‐growing importance of India, China, and the USA (Figure 4). The USA sees explosive growth in infected and removed populations around day 25, with infected cases overtaking India and China at around day 26 (Figure 5). Model results are displayed in rankings of countries most at risk according to the proportion of ACIs a country bears at a certain time (see Tables A2‐3, A2‐4 in the appendix).

**FIGURE 6:**
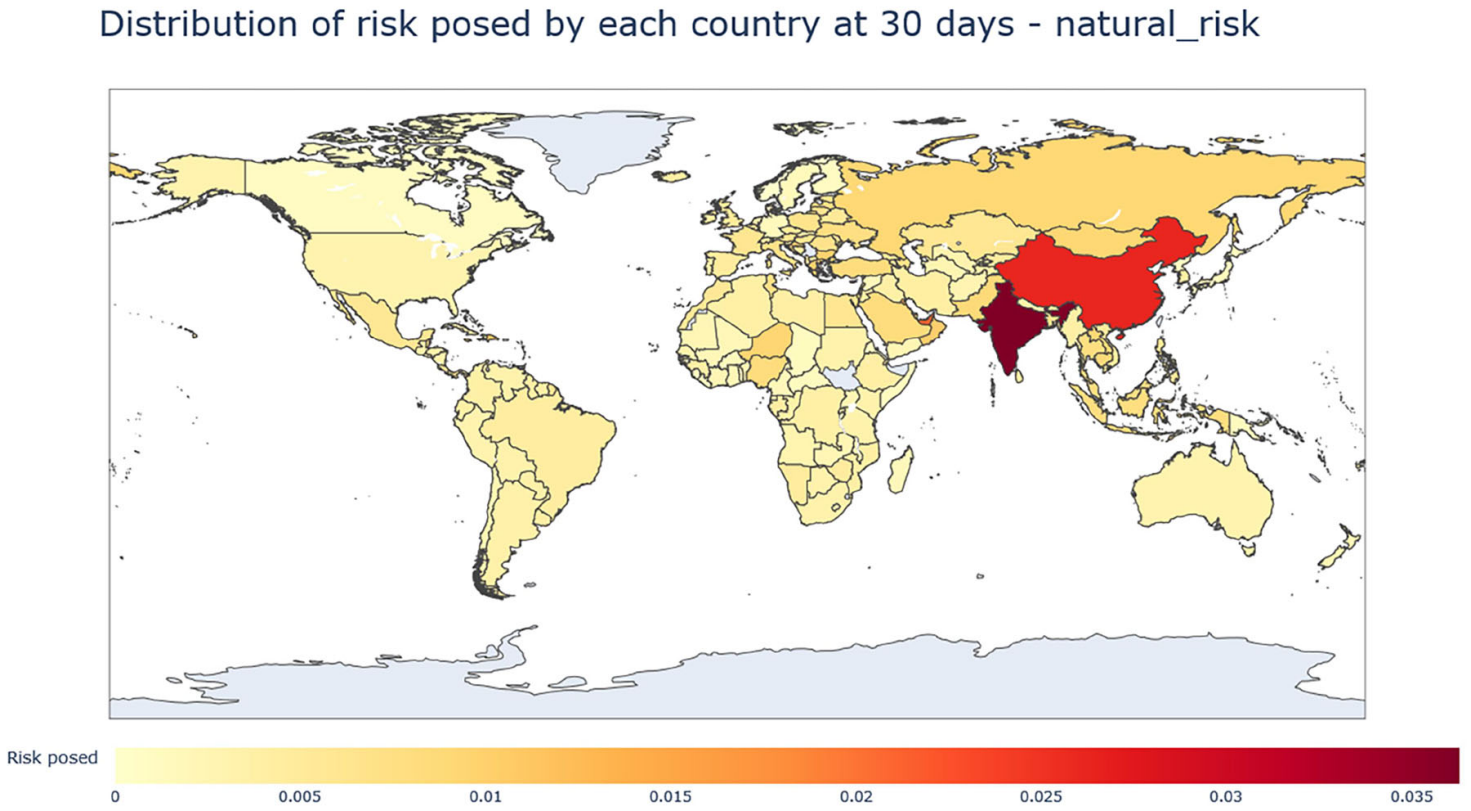
Distribution of risk posed by each country at day 30. India and China represent the largest risk posed, according to the used metric.

The risk that each country poses to the world was also studied as measured by the proportion of average cumulative infections that each country is responsible for. In order to obtain this number, we first obtain cumulative infections for each model run. Then, for each such model run, cumulative infections are multiplied by the EID origin risk of this run's country of origin, divided by the sum of EID origin risks. The resulting distribution of this measure of how much risk each country is responsible for at 30 days is shown in Figure 6.

Surprisingly, a few very small countries such as Bahrain, Seychelles, Cyprus, Palau, Malta, and Singapore are very high up on this list (see Appendix Table A3‐4). A potential explanation is that the Vulnerability Score from Moore et al. (2017) does not account directly for land area and total population, indicating that this risk measure misses an important factor. As such, the vulnerability scores likely give too much weight to very small countries since, in reality, more land area and population increase risk (*ceteris paribus*). These counter‐intuitive results concerning small countries may thus be an artifact of the fact that the vulnerability score from Moore et al. (2017) does not take into account area and population. This would suggest that a more accurate model would need to transform the EID origin potential so as to account for these two factors. A robustness check was run by re‐obtaining the results about ACIs by considering only relatively large countries (see Appendix 1—Large countries robustness check). It appears that model results are robust to removing small countries, with the overall ranking not changing significantly.

### 3.2 BSL 3+ and 4 lab leak model results

**FIGURE 7:**
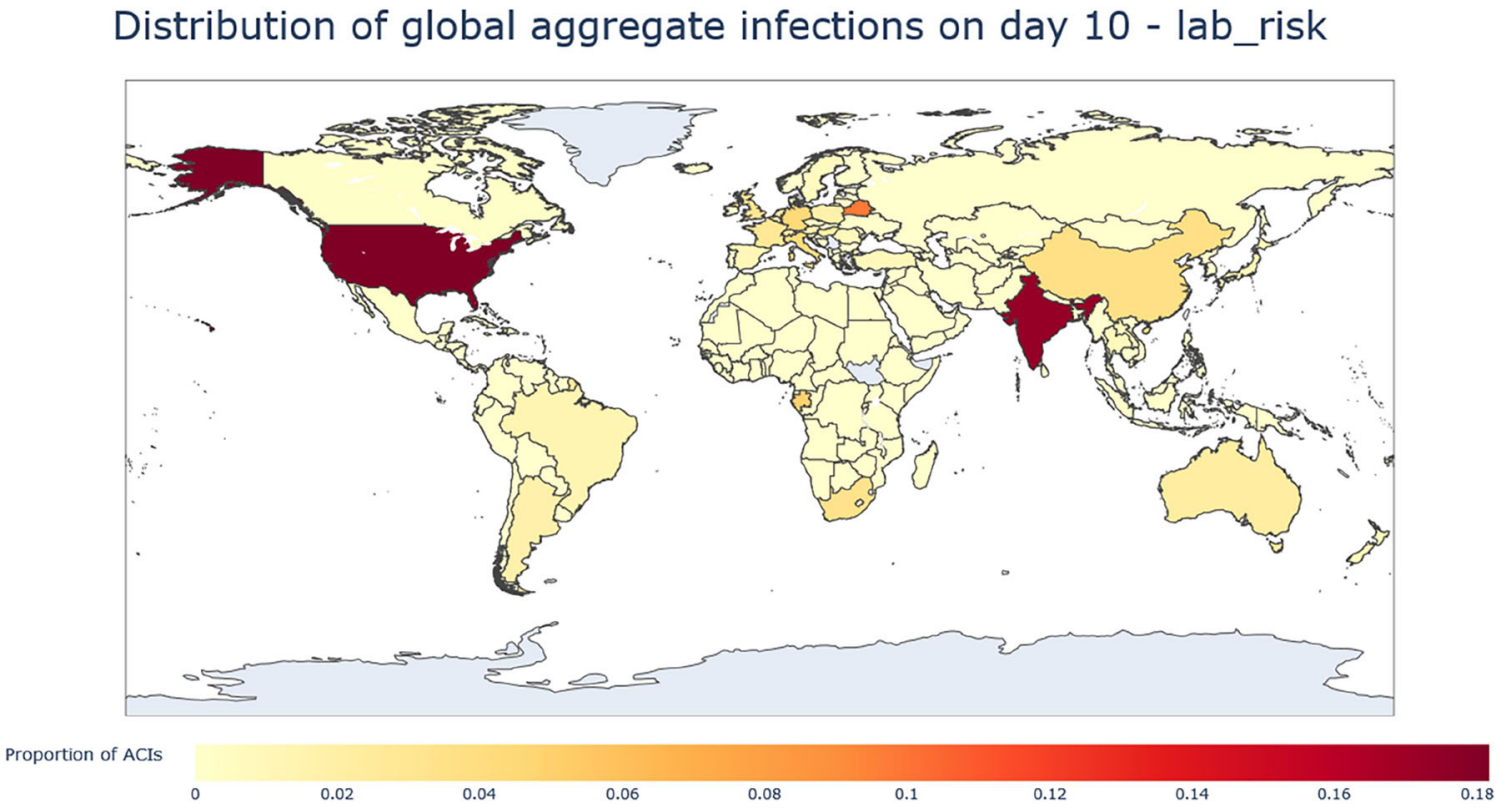
Heatmap of the ACIs from the lab leak origin at day 10, measured as proportion of ACIs. The map shows the USA and India as having the most ACIs.

**FIGURE 8:**
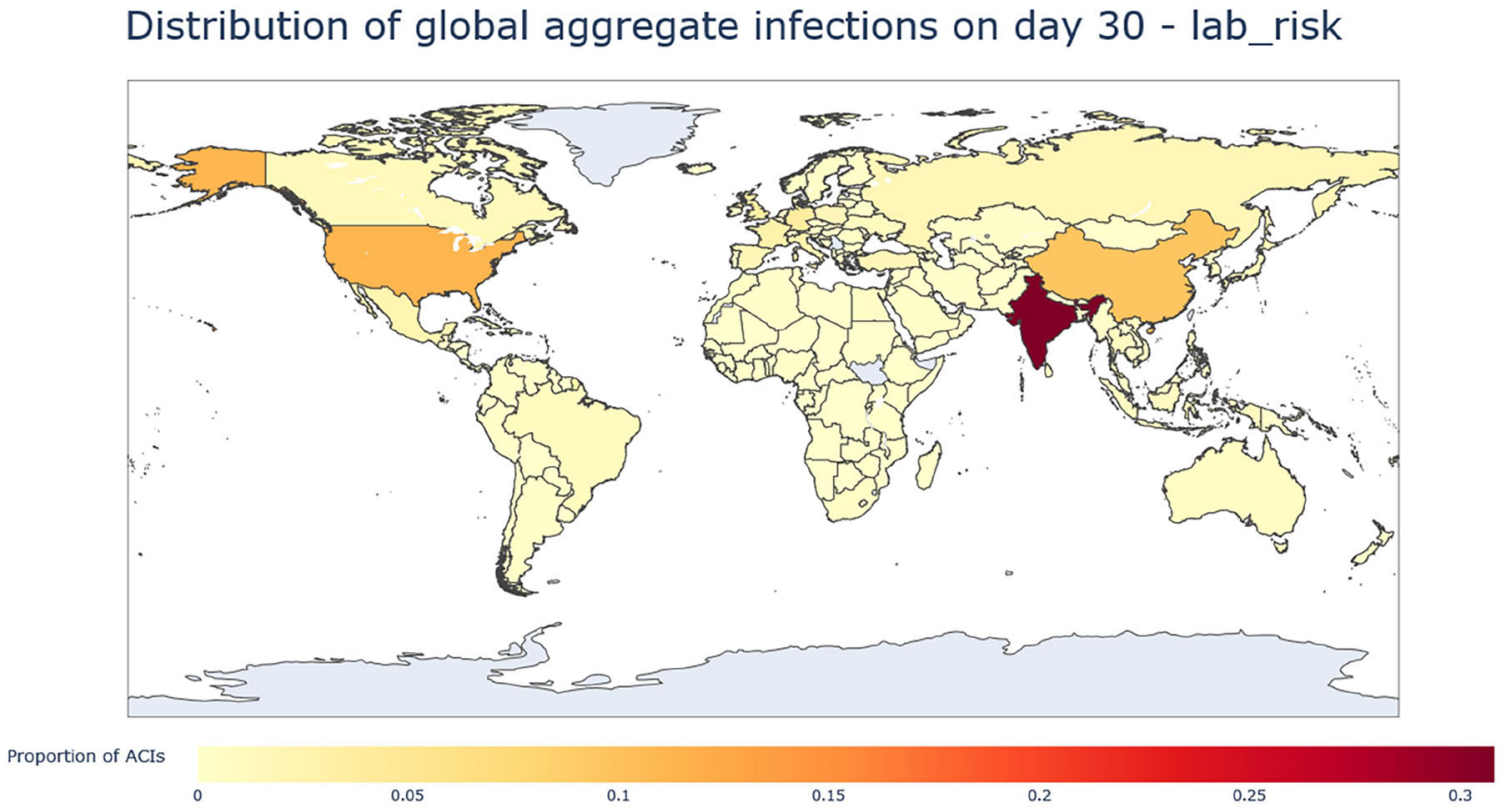
Heatmap of the ACIs from the lab leak origin at day 30, measured as proportion of ACIs. The map shows India as having by far the greatest proportion of ACIs; the USA's proportion has decreased from day 10, while China has increased.

**FIGURE 9:**
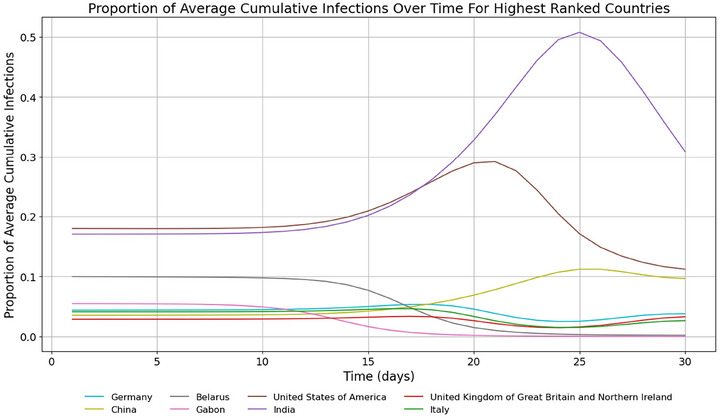
Proportion of ACIs by top‐ranking countries from days 1–30. Only countries in the top 5 at any time between day 1 and 30 are shown. The graph shows India as having by far the greatest proportion of ACIs.

Figures 7 and 8 show the proportion of ACIs in a country at days 10 and 30. The United States and India both start with the majority of ACIs at day 10, contributing ∼⅙ of the total at this point in time (Figure 9). Several other smaller countries also have a large amount of ACIs most notably Belarus and Gabon; this makes sense as Belarus possesses a large BSL 4 lab with area 1589 sqm and scores moderately on the GBBMS being 5th highest, while Gabon has two labs of (assumed average size) and possesses the worst GBBMS score of 0.92, which is over 3 times worse than the mean score.

**FIGURE 10:**
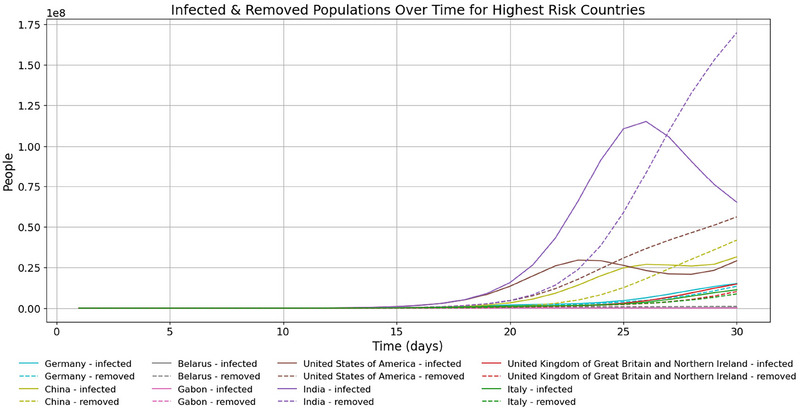
Time series of infected and removed populations. As above, the graph shows India's large population results in high numbers of infected and removed compared with other countries. Cases appear to have reached a first peak for India, with the USA and China starting second waves.

**FIGURE 11:**
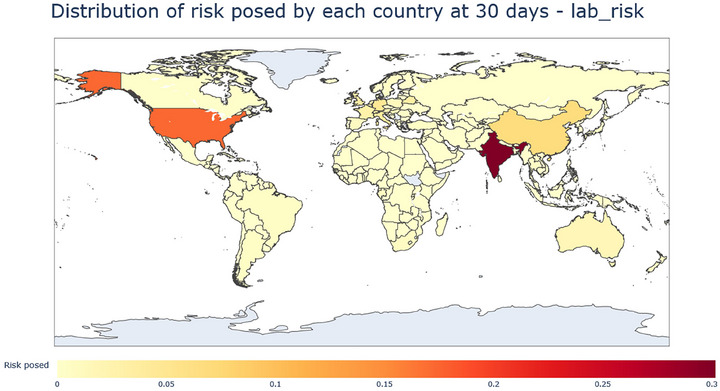
Distribution of risk posed by each country at day 30 from lab leaks. India represents the largest risk, followed by the USA and then China.

By day 30, India has taken over being the country with the highest proportion of ACIs, with the United States being the second highest. By day 30, China has the 3rd highest, having tripled from day 10, which could be explained by China's large population allowing much greater growth in the numbers of infected and removed (Figure 10).

Considering the total risk posed by each country at day 30 (Figure 11), which is measured by the share of average cumulative infections that each country is responsible for, India is the major contributor with a score of 0.30. The USA is second with a score of 0.112, and China is third with a score of 0.06 (Figure 9). Interestingly, Singapore scores quite high, being fourth overall (see Appendix Table A4‐3), which, given Singapore's relatively low lab risk, ranking 14, implies that it must be receiving a high number of infected and removed under a variety of pandemics when they are seeded in other countries. This implies that Singapore's location in global travel networks contributes to it being highly exposed to the risk of importing infections. Further analysis could examine key network metrics such as centrality and betweenness to identify which properties have the greatest influence on the system.

The lab leak model results appear to largely reflect the lab leak potential measure derived from the lab area and the GBBMS score of a country. One salient feature of the model result is that the risk stays concentrated in the USA and India from day 10 to day 30. Both countries start with a relatively large concentration of lab leak risk and also have large populations, allowing large numbers of infected and removed to be accrued at a high rate over the 30 days. Given the limitations of GBBMS, which only captures legislation requirements and does not consider implementation along with the inferred lab area, GBBMS data should be considered uncertain.

### 3.3 Comparison

Relative risk scores for EID and lab leak models cannot be compared directly, as the underlying risk metrics derived from EID vulnerability and GBBMS scores are not absolute measures of risk. That said, some high‐level insights can be gleaned, most notably the difference in how risk propagates through the network of countries and the influence of initial risk distribution.

The most salient difference between the models appears to be how the proportion of aggregate infections moves from day 10 to day 30. For lab leaks, labs are most highly concentrated in the USA and EU, whereas for the EID model, EID origin potential is highly concentrated in developing countries, most notably in Central Africa. This difference is important as in the lab leak model, the highest risk countries also function as major travel hubs, whereas in the EID model, impacted countries are less central in travel networks than the USA and EU.

## 4 DISCUSSION

The SIR model presented is best considered a preliminary investigation intended to guide future work. There are several important limitations, such as the country level of model abstraction, gaps in the datasets used, and assumptions pertaining to the EID and lab leak potential measures (see Appendix 5). Furthermore, the extensive literature on pandemic dynamics and associated modeling highlights the importance of many other factors not covered in our approach. These include seasonality of both disease transmission and travel patterns (Lai et al., 2022; Martinez, 2018), variations between short and long‐distance interactions in disease spread (Balcan et al., 2009), and heterogeneous disease spreading rates across different contexts and populations. These factors were not incorporated into the model to maintain simplicity and keep the focus on demonstrating how network interactions can be reconciled with origin risk. Nevertheless, limitations introduce significant uncertainty and limit the direct relevance of our findings for prioritization of pandemic preparedness interventions.

Solely focusing on the country level may overlook significant localized factors that heavily influence pandemic risk and offer high‐leverage locations for mitigation efforts. City‐level features such as population density, location of BSL 3+ and 4 labs in urban areas, and close proximity of highly connected travel nodes (major airports, ports, etc.) that facilitate rapid global spread are likely to make certain cities disproportionately impactful to EID and lab leak risk. Exploring EID and lab leak risk with models that use cities as nodes would capture critical features and allow more meaningful network analysis to be undertaken, improving model results and usefulness for prioritization.

Further work expanding or adapting the model to incorporate other important factors that influence risk would make valuable research. Promising directions include exploring alternative disease parameters, incorporating seasonal changes in infection rates, distinguishing between long and short‐distance interactions, and capturing how travel networks change in response to infections and travel restrictions.

The sensitivity analyses reveal important insights about model robustness (Appendices 1 and 2). The high rank‐bias overlap (0.777) when excluding small countries suggests our results are relatively insensitive to country size effects. The lower overlap (0.537) between different parameter sets indicates that specific country rankings are sensitive to disease characteristics. However, even with substantially different parameters, 24 of the top 30 countries remain consistent, suggesting stability in identifying the network positions most relevant to pandemic spread. This points to an important nuance in our findings: while the exact relative importance of countries may shift depending on disease characteristics, the insight about the need to consider both network position and origin risk remains valid. The model demonstrates that network effects can substantially alter risk landscapes regardless of specific parameter choices, underscoring the importance of incorporating travel patterns and connectivity when assessing pandemic risk. Future work, undertaking systematic sensitivity analysis across parameter ranges and alternative network structures, would be valuable to investigate how different classes of diseases might influence the relative importance of network positions.

If natural pandemics (represented by the EID model) represent the main source of risk, travel dynamics are highly influential. This is because the main countries of EID origin, as per EID vulnerability scores from Moore et al. (2017), are developing countries in Africa (Somalia, Central African Republic, Chad, South Sudan, Mauritania, and Angola). These countries are only minimally connected, and thus, the main channels of transmission from these poorly connected countries to the broader world are via other more connected countries. As a consequence, the main dynamic at play is the travel network rather than the distribution of origin, as demonstrated in the results by the shift in average cumulative infections from Central African countries to countries with significant travel. One interpretation of this result relevant to the prioritization of mitigation efforts is that the most connected countries are roughly both the most affected and the most important in global travel dynamics.

Conversely, if instead we assume lab leaks are the main source of risk, then the main origin countries are also quite globally connected countries (e.g., the USA, China, India, etc.). Thus, the distribution of pandemic origin risk is similar to the results at day 30. The list of highest risk countries then includes India and a few other countries that are not the richest but still heavily connected and with poor biosafety management, as captured by the GBBMS. India is especially of note, contributing ∼⅓ of the risk posed at day 30. This result is somewhat unsurprising given the presence of 5 BSL 3+ and 4 labs in the country and the second‐lowest biosafety management score of 0.77, behind only Gabon at 0.92, and being ∼2.7 times worse than the average score. All else equal, efforts to improve biosafety management practices in India may provide a good opportunity to reduce expected damages from lab leaks.

The concentration of risk in a small number of large countries (the USA, India, and China) at day 30 in both the EID and lab leak models indicates potentially high leverage points for pandemic risk mitigation. These countries, due to being central in the spatial network (derived from travel data) and/or contributing importantly to lab leak risk, have the power to significantly decrease the global risk of pandemic by implementing the right pandemic preparedness & response policies.

Public policies from the literature that appear consistent with our model results, potentially warranting further exploration, include:

- Deploying metagenomic sequencing in their main airports. Given that these countries are the nodes of the global traffic, this would increase the likelihood that spreading pathogens are identified rapidly by these airports, which can inform response actions (Li et al., 2023).
- Implementing well‐designed travel bans with precommitments in central countries may help avoid worst‐case contagion dispersion via air travel network nodes.

Our network analysis highlights that the policies of highly connected countries, as well as those with significantly high‐risk biolab setups, have the highest externalities. In the context of biolabs, which are clearer initiation sources, such externalities motivate stronger global concern with high‐containment facilities. Presently, the World Health Organization provides guidelines and biosafety best practices for BSL‐3 and BSL‐4 research (*Laboratory Biosafety Manual*, 2020); however, no global body exists to regulate their implementation. This leaves biosafety practices largely dependent on individual countries and researchers. Given the global externalities of pandemic risk, this lack of coordination is concerning. Indeed, current biosafety oversight varies substantially between jurisdictions, creating challenges for risk assessment and management (Lentzos et al., 2022).

Initiatives like the Global Biolabs project have highlighted both the rapid proliferation of BSL‐3 and BSL‐4 laboratories globally and the pressing need for harmonized safety standards and oversight mechanisms (Kobentz et al., 2023). A coordinated international framework for laboratory biorisk management could help standardize risk assessment methodologies, establish consistent safety protocols, and enable more accurate comparative risk analysis between facilities. Such standardization would be particularly valuable for the type of network‐based risk assessment presented in this paper, as it would provide more reliable input parameters for assessing laboratory‐origin risk factors. In summary, implementation of a global risk management regime, while politically challenging, would improve risk awareness and might contribute to risk mitigation.

While this paper focuses on natural emergence and laboratory accidents, it is worth noting that intentional release represents another potential pandemic origin pathway. Deliberate release from state actors, such as the Rhodesian Government in 1978–1979 during the Rhodesian insurgency and bioterrorism, or the Aum Shinrikyo attacks in Tokyo in the 1990s, demonstrate that actors with intent and capability exist. The decreasing costs of biotechnology processes and increasing accessibility of tools and knowledge create additional challenges for biosecurity (Esvelt, 2022). While various monitoring and control measures exist for critical materials and processes, the inherent dual‐use nature of many biotechnology advances makes comprehensive prevention challenging (Esvelt, 2022). Further research on biosecurity measures and governance frameworks could help address these concerns while supporting beneficial research.

These findings point toward the need for global coordination of pandemic risk mitigation efforts, ensuring that interventions account for both local and global risk.

## 5 CONCLUSION

The spatial network SIR model presented combines travel network data and two sources of potential pandemics (EID and lab leaks) to investigate early‐stage pandemic spread at the country level. Especially of interest is the BSL 3+ and 4 lab leak risk model, which synthesizes a novel lab leak potential score from recently collated BSL 3+ and BSL 4 lab data and country biosafety management practices. To the best of the authors’ knowledge, this represents a first attempt to develop such a model.

The integration of network analysis with origin risk assessment reveals insights that neither approach captures alone. Our findings highlight how global connectivity patterns can dramatically reshape risk distributions, whether from natural emergence or laboratory origins. While the model has important limitations, including parameter sensitivity and country‐level abstraction, it demonstrates the fundamental importance of considering both network position and origin risk in pandemic preparedness. This perspective suggests that effective risk reduction requires a more nuanced approach than simply targeting intervention efforts at predicted origin locations, pointing to the potential value of strategies that account for both origin risk and global connectivity patterns.

Results for both EID and lab leak models demonstrate that a handful of large, comparatively wealthy countries (the USA, India, and China) are likely to be heavily impacted by day 30 from pandemics initiated by either source. For EID‐initiated pandemics, these countries appear to import risk from EID origin countries concentrated in Central Africa (Nigeria, Democratic Republic of Congo, Equatorial Guinea, and Central African Republic), while for the lab leak model, these countries, especially the USA and India, generate the majority of risk. Consequently, mitigation efforts targeting these large, comparatively wealthy, highly connected countries may offer a better opportunity, all else equal, to reduce risk globally. Furthermore, the especially high externalities may be indicative of severe underinvestment in mitigation measures at such locations. The implication of this result runs somewhat counter to existing policy recommendations, such as Moore et al. (2017), which suggest focusing mitigation efforts on EID origin countries.

## AI TOOLS USAGE

This work utilized several AI tools in a supporting capacity. GitHub Copilot and Anthropic's Claude 3.7 were used as coding assistants during later versions of the developed model. Claude 3.7 was also used to help edit and refine several paragraphs in the manuscript for clarity and readability, particularly in the discussion section. The authors maintained full control over the research design, analysis, and conclusions, with AI tools serving purely as writing and coding assistants. All content was reviewed and verified by the authors.

## Figures and Tables

**FIGURE A1‐1:**
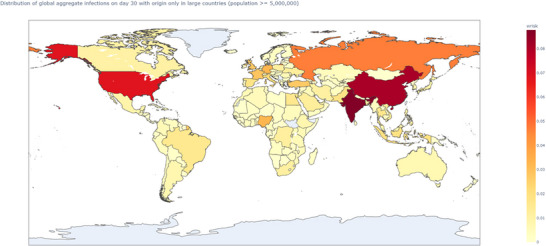
Distribution of global aggregate infections on day 30 with EID origin potential only considered for countries with populations greater than 5 million (i.e., small countries removed).

**FIGURE A1‐2:**
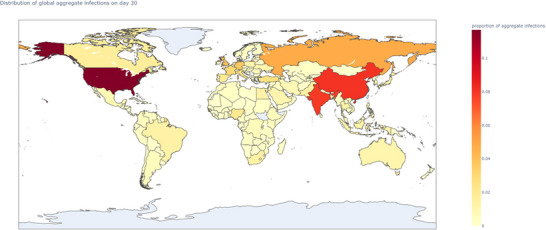
For easy comparison with Figure A1‐1, the distribution of global aggregate infections on day 30, including small countries.

**FIGURE A2‐1:**
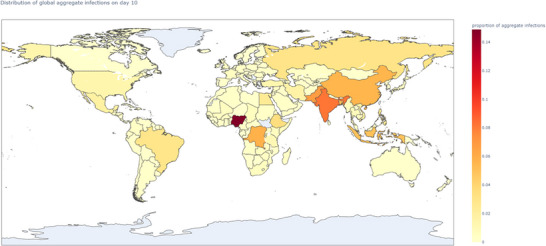
Heatmap of ACIs at 10 days.

**FIGURE A2‐2:**
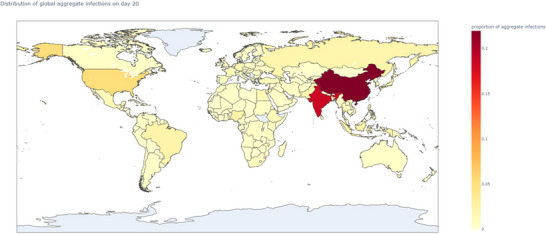
Heatmap of ACIs at 20 days.

**FIGURE A2‐3:**
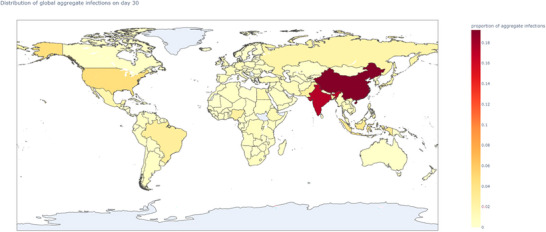
Heatmap of ACIs at 30 days.

**FIGURE A2‐4:**
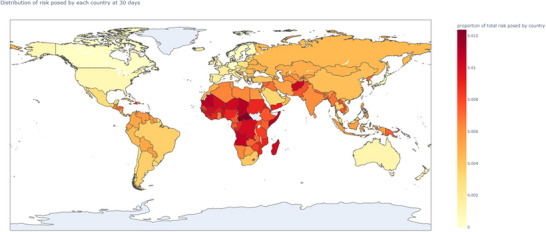
Heatmap of risk posed by each country (30 days).

**FIGURE A4‐1:**
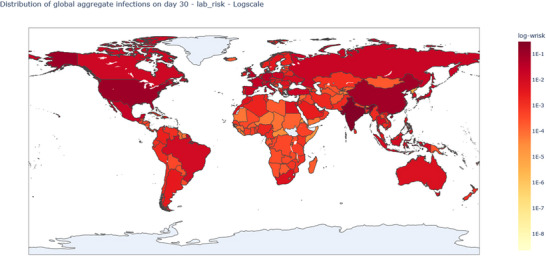
Heatmap of cumulative infections at day 10 ‐ Log scale.

**FIGURE A4‐2:**
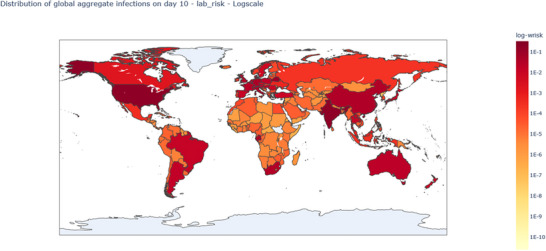
Heatmap of cumulative infections at day 30 ‐ Log scale.

## Data Availability

Code can be made available upon request from the authors.

## Footnotes

1 Removed refers to those removed from the pool of available hosts. The removed category captures recovered and assumed immune hosts or hosts who have died. This model misses more complex dynamics, such as decay of immunity.

2 Dataset can be explored interactively (European Commission, n.d.)

3 In the instance of synthetic pathogens, for example, pathogens developed through gain of function or other synthetic biology processes, determining plausible parameters would be even more complex, as such pathogens would not be bound by the proposed virulence trade‐off.

4 BSL 3+ or BSL 3 advanced labs are BSL 3‐designated labs that take on extra safety precautions to carry out research on riskier pathogens that do not require BSL 4 designation, for example, highly pathogenic influenza (Koblentz et al., 2023)

5 Data can also be interacted with through the Global Biolabs interactive map (Global Biolabs, n.d.)

6 Geometric mean was used over the median as lab area values indicated a lognormal distribution, and the sample size was relatively small, *n* = 51.

7 As risk of origin is relative (i.e., the model assumes a pandemic has happened and the country's relative contribution is determined), a linear scaling of risk assumption according to lab area will not result in absurd results (e.g., probability being greater than 1).

8 Cumulative infections are the sum of infected and recovered populations.

9 Rank‐Biased Overlap (RBO) measures the similarity of ranked lists of different lengths, accounting for both the relative position of items and the importance of higher ranks (Webber et al., 2010).

## APPENDIX 1 EID RISK—LARGE COUNTRIES ROBUSTNESS CHECK

In studying risk posed in the SIR model, we observed that small countries such as Cyprus appeared to account for a surprising share of the average cumulative infections. This seems to occur because the vulnerability scores by Moore et al. (2017) do not take population and land area into account (so the scores are higher than they perhaps should be for smaller countries). This could decrease the accuracy of results for the distribution of average cumulative infections over weighted model runs. Hence, we performed a robustness check by obtaining the distribution of average cumulative infections after removing the weights given to model runs that had small countries as origin (population < 5,000,000) (Table A1‐1, Figures A1‐1 and A1‐2).

**TABLE A1‐1:** Ranking comparison of top 30 average cumulative infections at 30 days for EID pandemic origin.

				Ranking top 30			
Ranking top 30				Large countries only			
All countries				(population >5,000,000)			
Country	I	R	Wrisk	Country	I	R	Wrisk
United States of America	18,546,533	8,172,089	0.1169	India	7,324,765	10,795,793	0.0878
China	11,242,643	7,233,951	0.0808	China	8,316,797	8,394,924	0.0809
India	9,627,360	8,398,861	0.0789	United States of America	10,259,859	3,833,911	0.0683
Russian Federation	6,971,863	3,883,675	0.0475	Russian Federation	6,229,289	3,450,057	0.0469
United Kingdom of Great Britain and Northern Ireland	5,580,502	3,171,135	0.0383	Germany	4,339,770	2,382,589	0.0326
Germany	5,477,127	2,878,455	0.0366	Nigeria	1,426,064	5,250,908	0.0323
France	4,546,969	2,273,980	0.0298	France	3,861,858	1,958,134	0.0282
Italy	3,848,005	2,071,099	0.0259	United Kingdom of Great Britain and Northern Ireland	3,818,273	1,916,715	0.0278
Turkey	3,801,441	1,983,450	0.0253	Turkey	3,671,375	1,892,930	0.0270
Nigeria	1,190,934	3,417,825	0.0202	Italy	2,981,529	1,535,198	0.0219
Spain	2,913,707	1,357,539	0.0189	Indonesia	1,554,446	2,438,618	0.0193
Canada	2,579,430	1,251,342	0.0168	Pakistan	1,199,333	2,631,954	0.0186
Pakistan	1,415,400	1,884,755	0.0144	Spain	2,523,549	1,254,697	0.0183
South Africa	1,870,041	1,384,918	0.0142	Thailand	2,007,320	1,409,778	0.0166
Egypt	1,906,643	1,209,710	0.0136	Egypt	1,855,836	1,414,260	0.0158
Indonesia	1,420,397	1,682,427	0.0136	Brazil	1,725,488	1,488,257	0.0156
Brazil	1,870,316	1,200,927	0.0134	Viet Nam	1,511,610	1,239,674	0.0133
Thailand	1,910,203	1,112,913	0.0132	Ukraine	1,521,621	1,116,846	0.0128
Japan	1,831,711	901,587	0.0120	Saudi Arabia	1,707,130	912,741	0.0127

We see that although positions do change in the ranking, the top countries are mostly still the same (Table A1‐1), differences can also be visually compared in Figures A1‐1 and A1‐2. The Rank Bias Overlap between these two rankings is of 0.777.

## APPENDIX 2 EID RISK—SIR MODEL WITH ALTERNATE PARAMETERS

We ran the SIR network model with different parameters: β = 3, γ = 0.1, and 500 initial infectives in each model run. See below the heatmaps, rankings, and ranking comparisons for EID origin sources (Figures A2‐1, A2‐2, A2‐3, A2‐4 and Tables A2‐1, A2‐2, A2‐3, A2‐4).

**TABLE A2‐1:** EID origin source average cumulative cases ranking 10 days.

Country	I	R	Wrisk
Nigeria	4,145,919	236,347	0.1488
India	2,326,475	85,764	0.0819
Pakistan	1,876,541	105,094	0.0673
Congo (Democratic Republic of the)	1,505,420	199,293	0.0579
China	1,568,671	57,602	0.0552
Bangladesh	1,380,338	85,845	0.0498
Indonesia	1,394,144	68,506	0.0497
Ethiopia	918,032	84,742	0.0341
Russian Federation	827,947	54,502	0.0300
Brazil	802,566	43,512	0.0287

**TABLE A2‐2:** EID origin source average cumulative cases ranking 20 days.

Country	I	R	Wrisk
China	89,994,180	13,191,954	0.2195
India	77,684,708	8,591,177	0.1835
United States of America	17,484,648	6,936,232	0.0520
Indonesia	10,487,156	14,538,559	0.0254
Nigeria	99,085,190	18,147,471	0.0249
Brazil	88,583,119	17,350,405	0.0225
Russian Federation	80,371,064	18,498,990	0.0210
Pakistan	81,544,959	10,823,690	0.0197
Japan	73,008,060	13,637,289	0.0184
Egypt	62,763,362	11,165,850	0.0157

**TABLE A2‐3:** EID origin source average cumulative cases ranking 30 days.

Country	I	R	Wrisk
China	56,331,753	82,587,644	0.1924
India	48,158,936	76,334,863	0.1733
United States of America	10,610,870	21,638,947	0.0447
Indonesia	105,246,360	143,212,612	0.0344
Brazil	77,258,914	121,619,191	0.0275
Pakistan	77,557,728	107,758,601	0.0257
Nigeria	69,548,385	110,084,743	0.0249
Bangladesh	65,025,285	89,633,237	0.0214
Russian Federation	56,508,639	86,732,176	0.0198
Mexico	51,696,688	74,100,586	0.0174

**TABLE A2‐4:** Top 10 EID origin source – risk posed (30 days) ranking.

Top 10 EID origin source – risk posed (30 days) ranking		
	Country	Risk posed
153	Central African Republic	0.0124
105	Somalia	0.0111
51	Mauritania	0.0111
17	Chad	0.0111
30	Angola	0.0108
0	Afghanistan	0.0104
13	Madagascar	0.0102
6	Niger	0.0102
19	Mali	0.0101
107	Congo (Democratic Republic of the)	0.0100

Moreover, we find that rankings differ somewhat from running this model with the parameters for SARS‐Cov2. The RBO for rankings of average cumulative infections for both models at 30 days is of 0.537. The intersection of the top 10 countries in the two rankings has only 5 countries. And the intersection of the top 30 countries has 24 countries. See below the top 30 countries (in order) according to model 3 with SARS‐Cov2 parameters and alternative parameters (Table A4‐1, A4‐2, A4‐3)5.

**TABLE A2‐5:** Ranking comparison of top 30 average cumulative infections at 30 days for EID pandemic origin.

Ranking top 30 SARS‐Cov2 parameters				Ranking top 30 alternate parameters			
Country	I	R	Wrisk	Country	I	R	Wrisk
United States of America	18,546,533	8,172,089	0.1169	China	56,331,753	82,587,644	0.192
China	11,242,643	7,233,951	0.0808	India	48,158,936	76,334,863	0.173
India	9,627,360	8,398,861	0.0789	United States of America	10,610,870	21,638,947	0.0447
Russian Federation	6,971,863	3,883,675	0.0475	Indonesia	105,246,360	143,212,612	0.0344
United Kingdom of Great Britain and Northern I…	5,580,502	3,171,135	0.0383	Brazil	77,258,914	121,619,191	0.0275
Germany	5,477,127	2,878,455	0.0366	Pakistan	77,557,728	107,758,601	0.0257
France	4,546,969	2,273,980	0.0298	Nigeria	69,548,385	110,084,743	0.0249
Italy	3,848,005	2,071,099	0.0259	Bangladesh	65,025,285	89,633,237	0.0214
Turkey	3,801,441	1,983,450	0.0253	Russian Federation	56,508,639	86,732,176	0.0198
Nigeria	1,190,934	3,417,825	0.0202	Mexico	51,696,688	74,100,586	0.0174
Spain	2,913,707	1,357,539	0.0189	Egypt	47,824,171	73,606,416	0.0174
Canada	2,579,430	1,251,342	0.0168	Philippines	39,765,597	63,913,126	0.0144
Pakistan	1,415,400	1,884,755	0.0144	Ethiopia	35,456,537	55,985,828	0.0136
South Africa	1,870,041	1,384,918	0.0142	Egypt	39,203,320	58,715,456	0.0135
Egypt	1,906,643	1,209,710	0.0136	Viet Nam	35,861,036	52,369,361	0.0130
Indonesia	1,420,397	1,682,427	0.0136	Germany	25,762,650	54,687,269	0.0114
Brazil	1,870,316	1,200,927	0.0134	Turkey	26,256,296	52,445,060	0.0112
Thailand	1,910,203	1,112,913	0.0132	Iran (Islamic Republic of)	31,695,608	45,120,114	0.0106
Japan	1,831,711	901,587	0.0120	Congo (Democratic Republic of the)	29,445,870	41,906,330	0.0097
Ukraine	1,599,607	1,067,765	0.0116	Thailand	25,427,170	43,959,908	0.0096
Poland	1,503,560	1,098,415	0.0114	United Kingdom of Great Britain and Northern I…	20,622,687	48,078,676	0.0092
Philippines	1,531,566	1,062,369	0.0114	France	18,900,094	45,208,465	0.0090
Australia	1,585,444	862,780	0.0112	Italy	19,124,931	42,369,647	0.0082
Saudi Arabia	1,543,855	962,415	0.0111	South Africa	20,188,236	36,449,460	0.0079
Bangladesh	1,169,127	1,355,682	0.0110	Tanzania, United Republic of	21,008,661	31,969,856	0.0074
Korea (Republic of)	1,517,391	681,190	0.0096	Korea (Republic of)	18,407,531	32,463,764	0.0070
Iran (Islamic Republic of)	1,214,696	863,596	0.0090	Myanmar	22,489,294	27,255,332	0.0070
Mexico	1,198,211	725,814	0.0084	Kenya	17,987,106	29,915,411	0.0066
United Arab Emirates	1,194,636	691,378	0.0083	Spain	14,842,927	31,391,680	0.0064
Viet Nam	1,053,624	614,564	0.0082	Colombia	16,962,058	26,930,406	0.0063

## APPENDIX 3 EXTENDED MODEL OUTPUTS FOR EID

Tables A3‐1, A3‐2, A3‐3

**TABLE A3‐1:** Day 10 top 10 countries according to proportion of ACIs.

Country	I	R	Proportion of ACIs
Nigeria	5952	1704	0.0215
Congo (Democratic Republic of the)	5885	1688	0.0212
Equatorial Guinea	4142	1364	0.0154
Somalia	3740	1084	0.0135
Central African Republic	3583	1066	0.0130
Chad	3370	976	0.0122
Dominican Republic	3300	961	0.0119
Angola	3221	927	0.0116
Mauritania	3187	951	0.0116

**TABLE A3‐2:** Day 20 top 10 countries according to proportion of ACIs.

Country	I	R	Proportion ACIs
Nigeria	1,202,070	456,668	0.0780
Congo (Democratic Republic of the)	727,079	374,007	0.0518
India	592,721	177,498	0.0362
Pakistan	542,605	204,129	0.0351
Bangladesh	419,783	165,663	0.0275
China	410,423	122,502	0.0251
Indonesia	382,577	134,817	0.0243
Ethiopia	348,668	160,521	0.0240
Russian Federation	301,238	116,703	0.0197
Egypt	260,116	121,677	0.0180

**TABLE A3‐3:** Day 30 top 10 countries according to proportion of ACIs.

Country	I	R	Proportion ACIs
United States of America	18,546,533	8,172,089	0.1169
China	11,242,643	7,233,951	0.0808
India	9,627,360	8,398,861	0.0789
Russian Federation	6,971,863	3,883,675	0.0475
United Kingdom of Great Britain and Northern Ireland	5,580,502	3,171,135	0.0383
Germany	5,477,127	2,878,455	0.0365
France	4,546,969	2,273,960	0.0298
Italy	3,848,005	2,071,099	0.0259
Turkey	3,801,141	1,983,450	0.0253
Nigeria	1,190,934	3,417,825	0.0202

**TABLE A3‐4:** Ranking of risk posed to the world by each country at day 30.

Country	Risk posed
India	0.0363
Maldives	0.0309
China	0.0262
United Arab Emirates	0.0212
Bahrain	0.0188
Seychelles	0.0165
Palau	0.0159
Cyprus	0.0139
Kuwait	0.0133
Malta	0.0118

## APPENDIX 4 EXTENDED MODEL OUTPUTS FOR BSL 3+ AND 4 LAB LEAKS

Figures A4‐1, A4‐2

**TABLE A4‐1:** Day 10 top 10 countries according to proportion of ACIs.

Country	I	R	Proportion of ACIs
United States of America	56,171	16,079	0.182
India	53,546	15,321	0.173
Belarus	30,041	8763	0.098
Gabon	14,879	4655	0.049
Germany	13,784	3952	0.045
Italy	12,775	3666	0.041
China	11,124	3183	0.036
South Africa	10,366	2975	0.034
United Kingdom of Great Britain and Northern Ireland	8965	2572	0.029
France	8504	2440	0.028

**TABLE A4‐2:** Day 30 top 10 countries according to proportion of ACIs.

Country	I	R	Proportion of ACIs
India	65,145,808	169,866,885	0.308
United States of America	29,133,532	56,164,609	0.112
China	31,538,291	41,894,582	0.096
Germany	15,177,979	13,519,508	0.038
United Kingdom of Great Britain and Northern Ireland	14,810,509	10,177,392	0.033
France	12,492,772	9,271,501	0.029
Italy	11,297,965	8,759,212	0.026
Mexico	11,850,272	5,280,472	0.022
Spain	7,832,971	6,418,677	0.019
Russian Federation	10,621,090	3,540,902	0.019

**TABLE A4‐3:** Ranking of risk posed to the world by each country at day 30 for lab leaks.

Country	Risk posed
India	0.3017
United States of America	0.1728
China	0.0674
Singapore	0.0471
Germany	0.0459
Italy	0.0394
United Kingdom of Great Britain and Northern Ireland	0.0335
Belarus	0.0332
Switzerland	0.0295
France	0.0280

## APPENDIX 5 MODEL LIMITATIONS

The model has limitations imposed by the geographic level of abstraction, simplifications of disease parameters, and assumptions relating to vulnerability scores for EID and how the GBBMS score and lab area map to lab leak risk.

The model does not include any mitigation attempts within the first 30 days; that is, countries do not attempt to adapt by implementing travel bans, quarantine, etc. Countries would be anticipated to respond to the outbreak once deaths or cases hit a certain level. A more advanced version of the model is currently being developed that will reduce travel to countries infected and removed above a certain level.

Country‐level resolution averages out important geographic and population characteristics, most notably intracountry population distribution and travel network characteristics, which are likely to strongly influence the risk potential for rapid increases in cases from EID outbreak and lab leak. Considering travel, major cities are often strongly tied into global air networks, acting as central travel hubs for many journeys. As such, they could rapidly spread pathogens globally. Expansion of the model to city‐level resolution and incorporation of city‐level travel network data would allow important characteristics of disease spread, such as population density of cities, colocation of BSL 3+ and 4 labs, and city‐level travel network data to be more readily investigated.

Intracountry population characteristics are likely to be especially important to the lab leak model due to the co‐location of BSL 3+ and 4 labs in high population density urban centers. For instance, the Murayama Annex, National Institute of Infectious Diseases (NIID) (BSL 4) is located ∼1.5 h from downtown Tokyo, which has a population of 39.8 million, while the 298 sqm of BSL 4 lab space of The Francis Crick Institute Containment 4 facility (formerly NIMR) is located ∼5 km from central London. These labs are likely to pose a far greater risk than an equivalent lab located in a rural or semirural area. Evidence for the significant influence of urban versus rural locations on BSL 3+ and 4 risk is provided by the agent‐based model developed by Merler et al. (Merler et al., 2013). The model reports a 3–5× decrease in the likelihood of a BSL 4 lab‐acquired influenza infection escaping detection and seeding an epidemic if a BSL 4 lab is rural as opposed to urban. The Global Biolabs data set contains an urban versus rural condition, which could be incorporated into the model to attempt to capture this dynamic.

Considering the above points, it is reasonable to assume that certain cities will contribute disproportionately high fractions of potential risk if they have large BSL labs in close proximity to large populations, high population density cities that act as global travel hubs. The current model is unable to inform prioritization to this level of resolution. Increasing model resolution to city‐level characteristics or using an alternative model approach able to capture such dynamics would make valuable future work and would potentially allow identification of key cities (and BSL labs) that contribute disproportionately large risk to the global risk landscapes.

The current model uses a single set of disease parameters for both EID and BSL lab escape to reduce complexity; as such, the model does not capture how differing virulence influences risk and how the virulence of the pathogen is distributed across different origin sources, that is, EID, BSL 3+ BSL 4. In reality, every model run (disease outbreak) is likely to be initiated by a different pathogen with different characteristics. The characteristics of the different origin sources would have different distributions of potential pathogens; for instance, two different BSL 4 labs would hold different pathogens, BSL 3+ labs would hold different types of pathogens as compared with BSL 4, and EIDs would yield different disease characteristics again. One might anticipate that, on average, pathogens from BSL 3+ and 4 labs that escape would be more virulent than EID, as by design, these labs would hold the most dangerous pathogens. Credibly determining the distribution of pathogen virulence between different sources would be a significant piece of work, though this would be valuable for improving future modeling efforts. A future version of the presented model could use such distributions to probabilistically select disease parameters based on historical data of known pandemic potential pathogens held in storage at BSL 3+ and 4 labs, and then running the SIR model with selected disease parameters before aggregating all runs.
